# Younger age and prior graft failure are associated with increased risk of ACL reinjury: Graft survival and contralateral ACL outcomes at a median 10.6‐year follow‐up after primary hamstring autograft reconstruction

**DOI:** 10.1002/jeo2.70828

**Published:** 2026-06-29

**Authors:** Diego Alarcón Perico, Jorge Rojas Lievano, Sergio González, German Carrillo Arango, Gamal Zayed, Klaus Mieth Alviar

**Affiliations:** ^1^ Department of Orthopedics and Traumatology Hospital Universitario Fundación Santa Fe de Bogotá Bogotá Colombia; ^2^ Department of Orthopedics and Traumatology Hospital Serena del Mar Cartagena Colombia; ^3^ School of Medicine Universidad de Los Andes Bogotá Colombia

**Keywords:** anterior cruciate ligament, contralateral ACL injury, failure incidence, graft survival, long‐term follow‐up, risk factors

## Abstract

**Purpose:**

To evaluate graft survival and contralateral anterior cruciate ligament (ACL) injury after primary hamstring autograft reconstruction and identify factors associated with these outcomes at a minimum follow‐up of 7 years.

**Methods:**

This retrospective cohort study included 415 randomly selected patients who underwent primary single‐bundle hamstring tendon ACL reconstruction between 2005 and 2014. Patients were followed for a median of 10.6 years (range, 7.6–17.1). Graft failure was defined as revision reconstruction, clinically or imaging‐confirmed rupture or patient‐reported characteristic instability in a prespecified worst‐case approach. Kaplan–Meier analysis was used to estimate graft survival and contralateral ACL survival. Cox proportional hazards models were used to identify factors associated with graft failure and contralateral injury. Prior graft failure in the index knee was modelled as a time‐varying covariate in the contralateral injury analysis.

**Results:**

The 10‐year graft survival probability was 86.6% (95% confidence interval [CI], 82.8%–89.5%). Age 21 years or younger was associated with a higher hazard of graft failure (adjusted hazard ratio [aHR], 2.62; 95% CI, 1.49–4.61; *p* < 0.001). Concomitant chondral lesions were associated with a lower hazard of graft failure (aHR, 0.29; 95% CI, 0.11–0.73; *p* = 0.009). The 10‐year contralateral ACL survival probability was 95.5% (95% CI, 92.9%–97.1%). Prior graft failure in the index knee was associated with contralateral ACL injury (aHR, 3.25; 95% CI, 1.00–10.53; *p* = 0.050).

**Conclusion:**

At a median follow‐up of 10.6 years after primary hamstring autograft ACL reconstruction, graft survival was 86.6% and contralateral ACL survival was 95.5%. Younger age was associated with a higher hazard of graft failure, whereas concomitant chondral lesions were associated with a lower hazard of graft failure. Prior graft failure in the index knee was associated with subsequent contralateral ACL injury.

**Level of Evidence:**

Level III.

## Graphical abstract

Younger age (≤21 years) and prior graft failure independently predicted anterior cruciate ligament (ACL) reinjury risk in both knees at a median 10.6‐year follow‐up after hamstring autograft reconstruction.

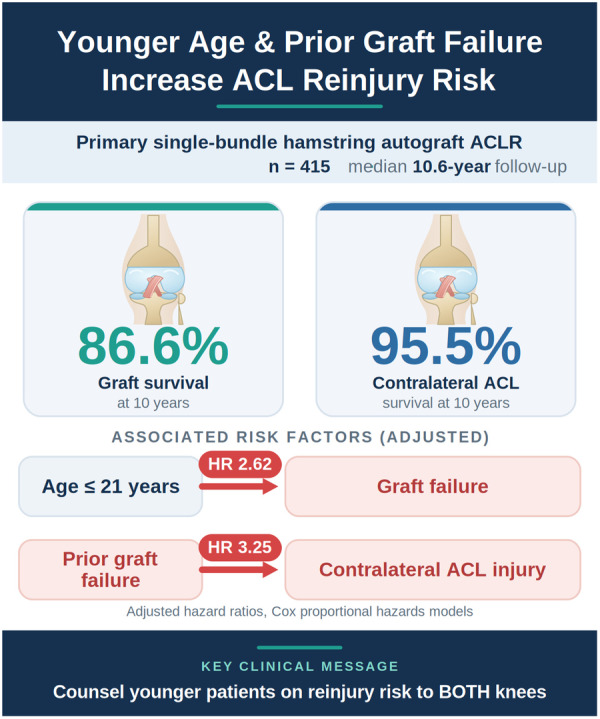

**Abbreviations**
ACL: anterior cruciate ligament
aHR: adjusted hazard ratio
BMI: body mass index
BPTB: bone–patellar tendon–bone (referenced in exclusion criteria)
CI: confidence Interval
HR: hazard ratio
IQR: interquartile range
IRB: institutional review board
REDCap: Research Electronic Data Capture
SANE: Single Assessment Numeric Evaluation
SD: standard deviation

## INTRODUCTION

Anterior cruciate ligament (ACL) injury is a common condition, particularly in active individuals, and ACL reconstruction is performed to restore knee stability and facilitate return to physical and sports activities. In the United States, ACL reconstruction has an estimated annual incidence of 43.5 per 100,000 person‐years [11]. Although ACL reconstruction is generally successful, with reported satisfaction rates ranging from 75% to 97% [14], graft failure and contralateral ACL injury remain important complications after surgery.

Reported rates of ACL graft failure vary substantially across studies. In the largest study to date, including 104,255 patients followed for up to 10 years, the incidence of graft rupture was 3.2%, with most failures occurring within the first 5 postoperative years [1]. Other case series and registry‐based cohorts have reported graft failure rates ranging from 3% to 20% [2, 4, 7, 25, 27, 28]. This variability likely reflects differences in study design, duration of follow‐up, definitions of graft failure and completeness of follow‐up. In addition, there is considerable heterogeneity across studies regarding factors associated with graft failure, including demographic characteristics, surgical variables and postoperative events.

Contralateral ACL injury is also a relevant long‐term concern after reconstruction. Previous studies have reported contralateral ACL injury in 5%–15% of patients during follow‐up [5, 16, 17, 25]. Although several factors have been proposed, including age, family history, activity level, joint laxity, intercondylar notch morphology and native ACL size, the factors associated with contralateral ACL injury remain incompletely understood [9, 16, 35].

The present study aimed to evaluate both outcomes within the same cohort. It was hypothesized that graft failure in the index knee would be associated with subsequent contralateral ACL injury.

## METHODS

### Study design and eligibility

This retrospective cohort study evaluated patients who underwent primary ACL reconstruction at a tertiary referral centre between 2005 and 2014. The study was approved by the institutional review board (IRB). Inclusion criteria were: (1) primary ACL reconstruction performed at the study institution between 2005 and 2014 and (2) availability of complete medical records. Exclusion criteria were: (1) previous ACL reconstruction in the affected knee; (2) osteoarthritis of Kellgren–Lawrence Grade 3 or greater in any compartment; (3) incomplete medical records or (4) refusal to participate. After application of these criteria, 1006 patients were eligible for sampling.

### Sampling strategy and follow‐up

The study was originally designed to evaluate outcomes after a minimum follow‐up of 5 years. However, because approximately 2 years elapsed between study conception and the start of data collection owing to IRB approval and preparation for telephone‐based follow‐up, all contacted patients had a minimum follow‐up of 7 years at the time of data collection. Telephone follow‐up was performed between July and August 2022. Median follow‐up was 10.6 years (interquartile range [IQR], 9.0–12.3; range, 7.6–17.1).

A target sample of 502 patients, corresponding to 50% of the eligible cohort, was selected to balance statistical precision with the logistical demands of telephone‐based follow‐up and to allow for anticipated post‐survey exclusions. Patients were sampled using simple random sampling. A random number table generated in Microsoft Excel was used to determine the order in which patients were contacted. All telephone interviews were conducted by a trained research assistant who was not involved in the index surgical procedures. If a selected patient could not be reached or declined participation, the next patient on the randomized list was contacted. This process continued until 502 patients completed the survey; 96 selected patients could not be reached or declined participation and were replaced in this manner.

### Final analytical cohort

**Figure 1:**
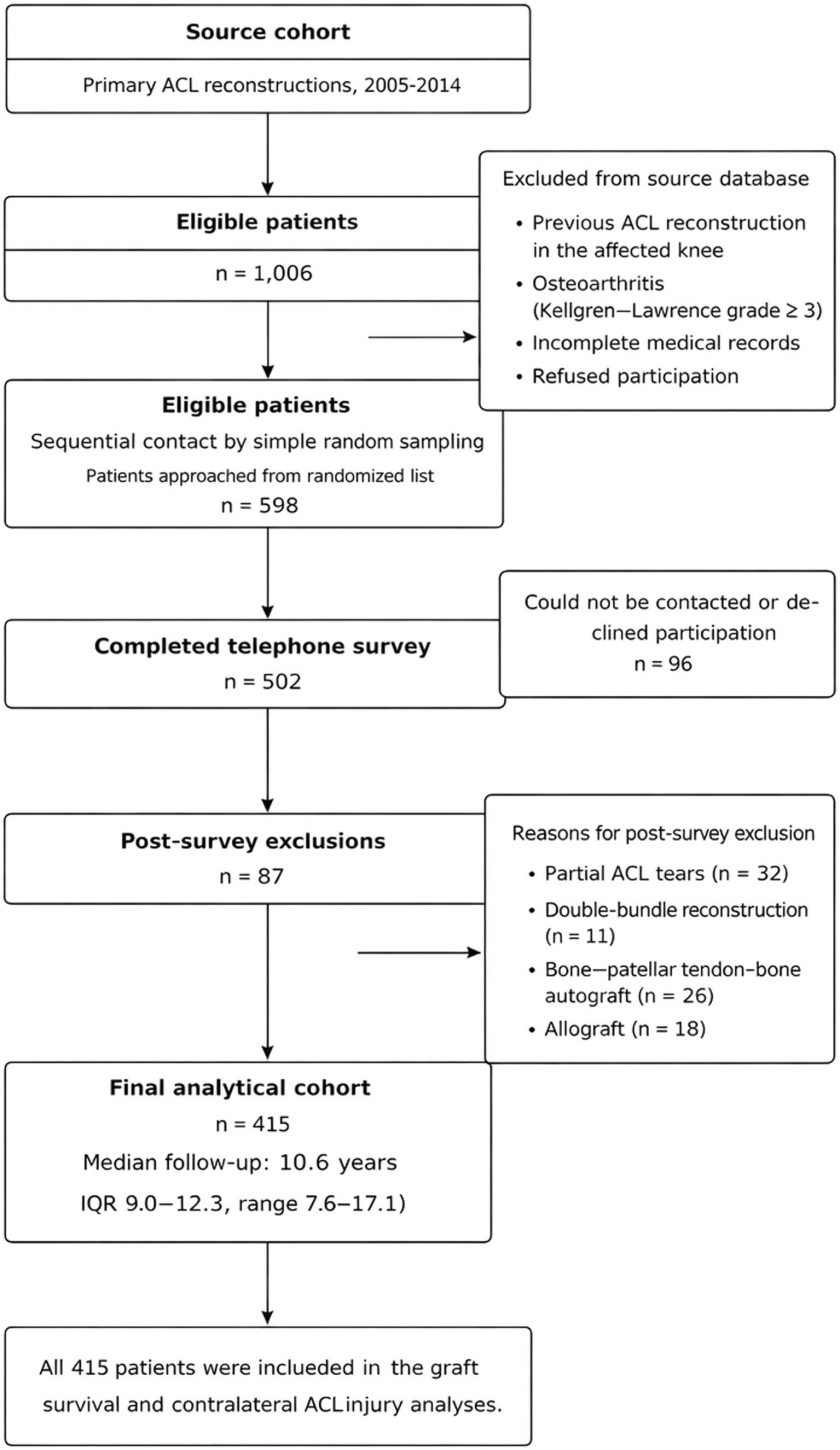
Study flow diagram. ACL, anterior cruciate ligament.

To create a homogeneous analytical cohort limited to single‐bundle hamstring tendon autograft reconstruction, 87 of the 502 surveyed patients were excluded after interview review: 32 because of partial ACL tears, 11 because of double‐bundle reconstruction, 26 because of bone–patellar tendon–bone autograft and 18 because of allograft use. When more than one exclusion criterion was present, the primary reason for exclusion was recorded. The final analytical cohort consisted of 415 patients (Figure 1).

### Patient‐reported follow‐up assessment

All included patients completed a structured questionnaire developed for this study. Before use, the questionnaire was pilot tested in 10 patients and demonstrated satisfactory comprehension and acceptance. The questionnaire assessed graft survival, timing of any subsequent injury, further surgery in either knee, contralateral ACL injury, preinjury and postoperative sports participation using the Tegner activity scale [33] and knee function using the Single Assessment Numeric Evaluation (SANE) score [29]. Information obtained during the telephone interview was cross‐checked against the medical record when available. Because prospective preinjury Tegner data were not available, preinjury activity level was assessed retrospectively at the time of follow‐up.

### Definition of graft failure and contralateral ACL injury

Graft failure and contralateral ACL injury were defined by one of the following criteria: (1) revision ACL reconstruction in the index knee or primary ACL reconstruction in the contralateral knee, whether performed at the study institution or elsewhere; (2) confirmation of injury by clinical examination and/or magnetic resonance imaging at the study institution or (3) patient‐reported characteristic ACL injury, defined as an acute episode of knee instability after a pivoting, decelerating or landing mechanism, with or without an audible pop and subsequent effusion, in the absence of clinical or imaging confirmation.

In the third scenario, the event was classified as graft failure or contralateral injury according to a prespecified worst‐case approach for survival analysis. This approach was chosen to reduce underascertainment of clinically meaningful late events in a long‐term retrospective cohort, particularly when injuries may have been evaluated or treated at external facilities. However, events classified exclusively on the basis of patient report may not always represent true structural graft rupture or contralateral ACL tear. Data from the telephone survey and medical records were managed using Research Electronic Data Capture.

Meniscal pathology was recorded as the presence or absence of a meniscal lesion for the full cohort. Detailed tear morphology and meniscal treatment were not available in a standardized manner across the study period, and these variables therefore were not suitable for stratified analysis.

### Surgical technique and rehabilitation

All procedures in the analytical cohort were performed by three fellowship‐trained knee surgeons using a standardized arthroscopic single‐bundle technique with an anteromedial portal for femoral tunnel drilling. All patients received hamstring tendon autograft using semitendinosus and gracilis tendons, with a minimum graft diameter of 8 mm according to institutional surgical practice. Fixation was tailored to the individual case and included interference screws, post screws and/or tenodesis as indicated. No lateral extra‐articular tenodesis or anterolateral tenodesis procedures were performed in this cohort.

All procedures were performed on an outpatient basis. Immediate full weight‐bearing without knee immobilization was permitted. Supervised physical therapy was initiated approximately 5 days after surgery, followed by a structured rehabilitation programme. Return to contact sports was permitted after a minimum of 12 months. No standardized criteria‐based return‐to‐sport protocol was used during the study period; clearance was based on surgeon assessment.

### Statistical analysis

Continuous variables are presented as mean ± standard deviation, and categorical variables as number and percentage. Non‐normally distributed variables, including Tegner activity level and time from injury to surgery, are presented as median, IQR and range. A sample size calculation based on Greenwood's formula for the precision of Kaplan–Meier estimates [6], assuming a 10‐year graft survival probability of 90%, a 95% confidence interval (CI) half‐width of ±3%, and finite population correction for a source population of 1006 patients, indicated that at least 279 patients would be required. The final analytical cohort of 415 patients exceeded this threshold.

Graft survival and contralateral ACL survival were estimated using the Kaplan–Meier method, and survival probabilities were reported at 2, 5 and 10 years. Annualized failure rates were calculated by dividing the number of events by the cumulative person‐years of follow‐up.

Univariable Cox proportional hazards regression was used to evaluate associations between candidate variables and each outcome. Variables with a *p* value < 0.25 in univariable analysis were entered into multivariable Cox regression models. Covariates were removed if they were not statistically significant and did not act as confounders, defined as a change greater than 15% in any remaining parameter estimate after removal. Candidate variables included age (≤21 years vs. >21 years), sex, body mass index (BMI), meniscal lesion, chondral lesion, preinjury Tegner activity level, weekly sports frequency and time from injury to reconstruction. For the contralateral injury model, graft failure in the index knee was entered as a time‐varying covariate to account for the time‐dependent nature of this exposure. Because only 18 contralateral ACL injuries occurred, the multivariable model for this outcome was considered exploratory.

The proportional hazards assumption was assessed by visual inspection of log‐log survival plots and was met for the final models. Model discrimination was assessed using Harrell's concordance index. Missing covariate data were handled by complete‐case analysis. Statistical significance was defined as *p* < 0.05. All analyses were performed using Stata version 17 (StataCorp).

## RESULTS

### Baseline characteristics

A total of 415 patients were included in the final analytical cohort and followed for a median of 10.6 years (IQR, 9.0–12.3; range, 7.6–17.1). The mean age at surgery was 29.8 ± 10.8 years (range, 13–61 years), and 123 patients (29.6%) were aged 21 years or younger. There were 332 male patients (80.0%) and 83 female patients (20.0%). Concomitant meniscal lesions were present in 255 of 414 patients (61.4%), and concomitant chondral lesions were present in 124 of 413 patients (29.9%). Additional baseline characteristics are summarized in Table 1.

**Table 1:** Patient demographics and baseline characteristics (*N* = 415).

Variable	Value
Age at surgery, years, mean ± SD	29.8 ± 10.8
≤21 years, *n* (%)	123 (29.6)
>21 years, *n* (%)	292 (70.4)
Sex, *n* (%)	
Male	332 (80.0)
Female	83 (20.0)
BMI, kg/m^2^, mean ± SD^a^	24.5 ± 3.0
Affected side, *n* (%)	
Right	227 (54.7)
Left	188 (45.3)
Time from injury to surgery, *n* (%)	
<3 weeks	223 (53.7)
3–12 weeks	140 (33.7)
>12 weeks	49 (11.8)
Concomitant meniscal lesion, *n* (%)^b^	255 (61.4)
Concomitant chondral lesion, *n* (%)	124 (29.9)
Pre‐injury Tegner activity level, median (IQR)	8 (6–8)
Weekly sport frequency, median (IQR)	3 (2–3)
Pre‐injury sport participation, *n* (%)	396 (95.4)
Follow‐up, years, mean ± SD (range)	10.7 ± 1.9 (7.6–17.1)

Abbreviations: ACL, anterior cruciate ligament; BMI, body mass index; IQR, interquartile range; SD, standard deviation.

^a^ Available for 411 patients.

^b^ Meniscal lesion was recorded as the presence of any documented meniscal injury. Detailed tear pattern and meniscal treatment were not available in a standardized manner across the study period.

### ACL graft survival

**Figure 2:**
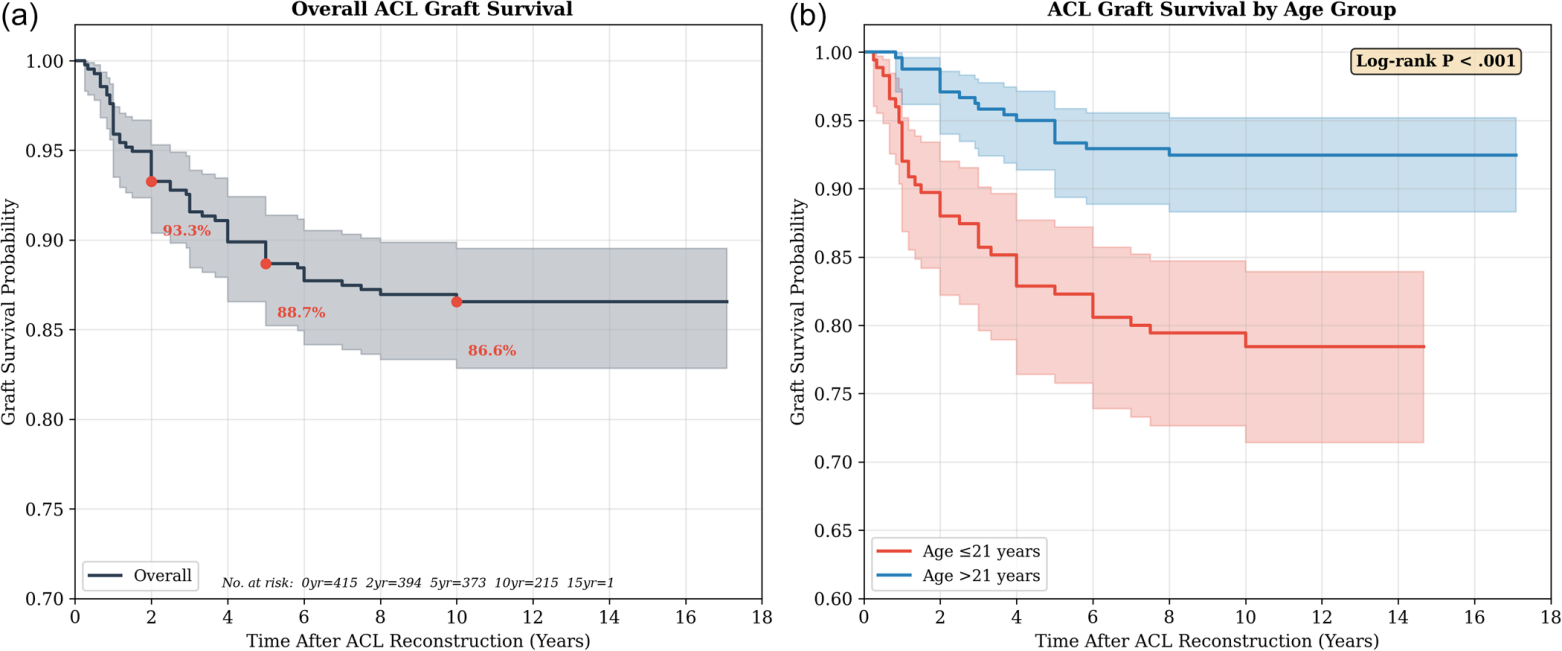
Kaplan–Meier survival analysis of ACL graft survival. (a) Overall ACL graft survival probability over time. Survival estimates at 2, 5 and 10 years were 93.3%, 88.7% and 86.6%, respectively. The shaded area represents the 95% confidence interval. (b) ACL graft survival stratified by age at the time of surgery (≤21 years vs. >21 years). Patients aged ≤21 years demonstrated significantly lower graft survival compared with patients aged >21 years (log‐rank *p* < 0.001). Shaded areas represent 95% confidence intervals for each group. ACL, anterior cruciate ligament.

Fifty‐five patients (13.2%) experienced graft failure during follow‐up at a mean of 35.1 ± 27.0 months after reconstruction (median, 24.0; range, 3–120 months). The annualized graft failure rate was 1.38% per knee‐year over 3985 person‐years of observation. Kaplan–Meier graft survival was 93.3% (95% CI, 90.4%–95.3%) at 2 years, 88.7% (95% CI, 85.2%–91.4%) at 5 years and 86.6% (95% CI, 82.8%–89.5%) at 10 years (Figure 2a). Patients aged 21 years or younger had lower graft survival than patients older than 21 years (Figure 2b).

**Figure 3:**
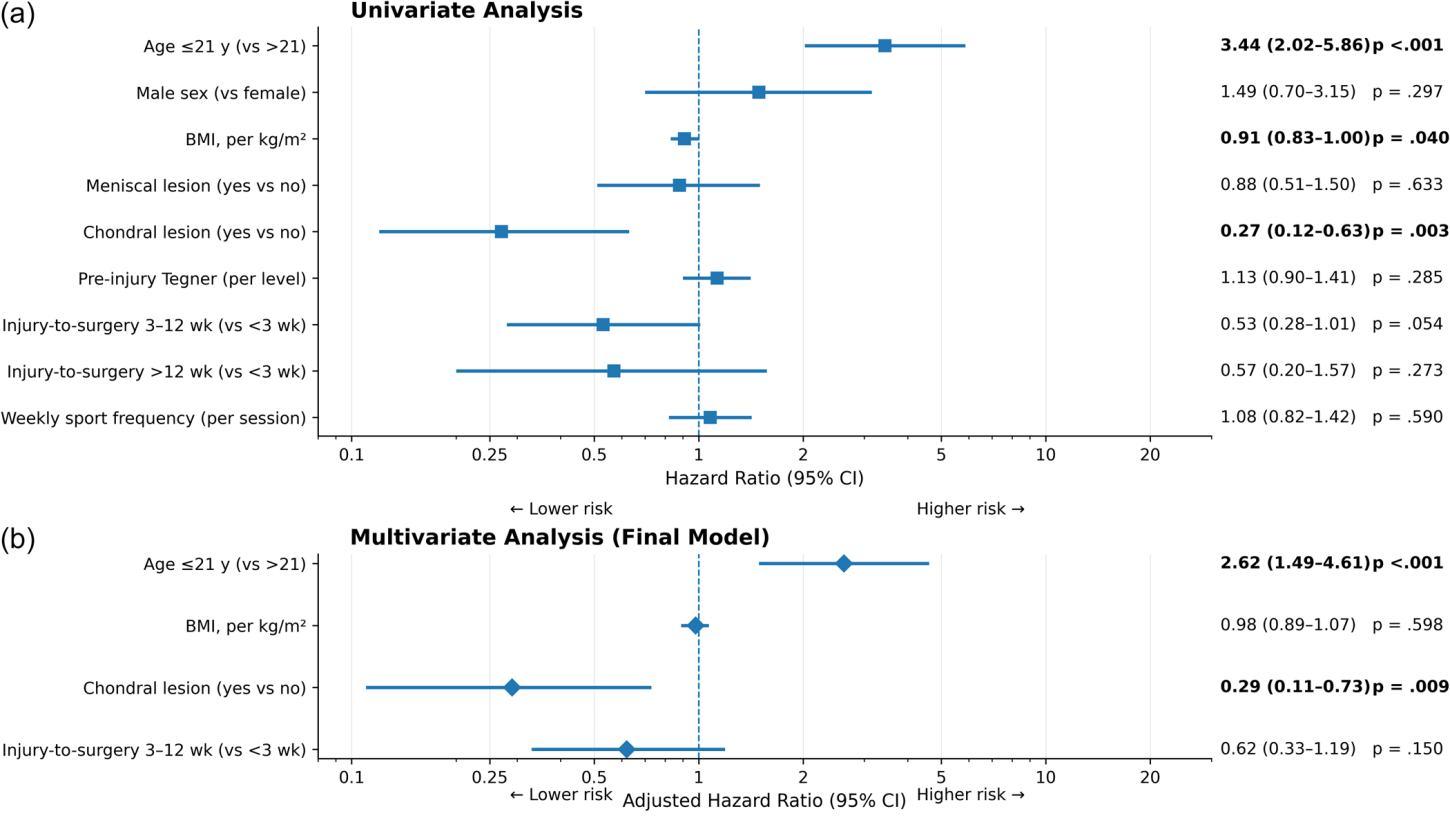
Forest plot of Cox regression analyses for risk factors associated with ACL graft failure after primary ACL reconstruction. (a) Univariate HRs and 95% CIs. (b) Multivariate model with aHRs and 95% CIs. The dashed vertical line indicates HR = 1.0. Bold text denotes statistically significant associations. ACL, anterior cruciate ligament; aHRs, adjusted hazard ratios; BMI, body mass index; CI, confidence interval; HRs, hazard ratios.

In univariable Cox regression, age 21 years or younger, lower BMI and concomitant chondral lesions were associated with graft failure (Figure 3a). In multivariable analysis, age 21 years or younger remained associated with a higher hazard of graft failure (adjusted hazard ratio [HR], 2.62; 95% CI, 1.49–4.61; *p* < 0.001), whereas concomitant chondral lesions remained associated with a lower hazard of graft failure (adjusted HR, 0.29; 95% CI, 0.11–0.73; *p* = 0.009) (Figure 3b). Sex, meniscal lesion status, preinjury Tegner activity level, weekly sports frequency and time from injury to reconstruction were not associated with graft failure.

### Contralateral ACL injury

**Figure 4:**
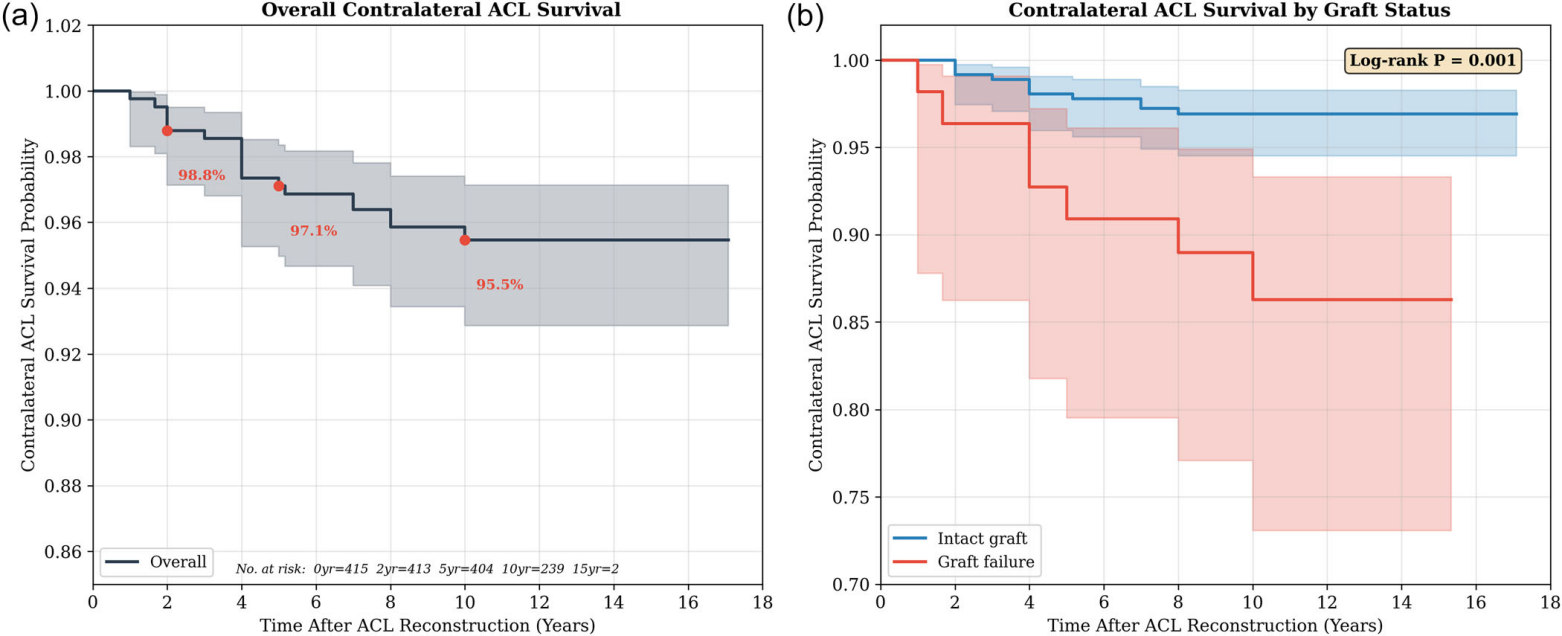
Kaplan–Meier survival analysis of contralateral ACL survival. (a) Overall contralateral ACL survival probability over time. Survival estimates at 2, 5 and 10 years were 98.8%, 97.1% and 95.5%, respectively. The shaded area represents the 95% confidence interval. (b) Contralateral ACL survival stratified by index ACL graft failure status. Patients who experienced graft failure had significantly lower contralateral ACL survival compared with those without graft failure (log‐rank *p* < 0.001). Shaded areas represent 95% confidence intervals for each group. ACL, anterior cruciate ligament.

Eighteen patients (4.3%) sustained a contralateral ACL injury during follow‐up at a mean of 54.6 months after reconstruction (median, 48.0; range, 12–120 months). The annualized contralateral ACL injury rate was 0.42% per knee‐year over 4315 person‐years of observation. Kaplan–Meier contralateral ACL survival was 98.8% (95% CI, 97.1%–99.5%) at 2 years, 97.1% (95% CI, 95.0%–98.3%) at 5 years and 95.5% (95% CI, 92.9%–97.1%) at 10 years (Figure 4a). Patients who experienced graft failure had lower contralateral ACL survival than those without graft failure (Figure 4b).

**Figure 5:**
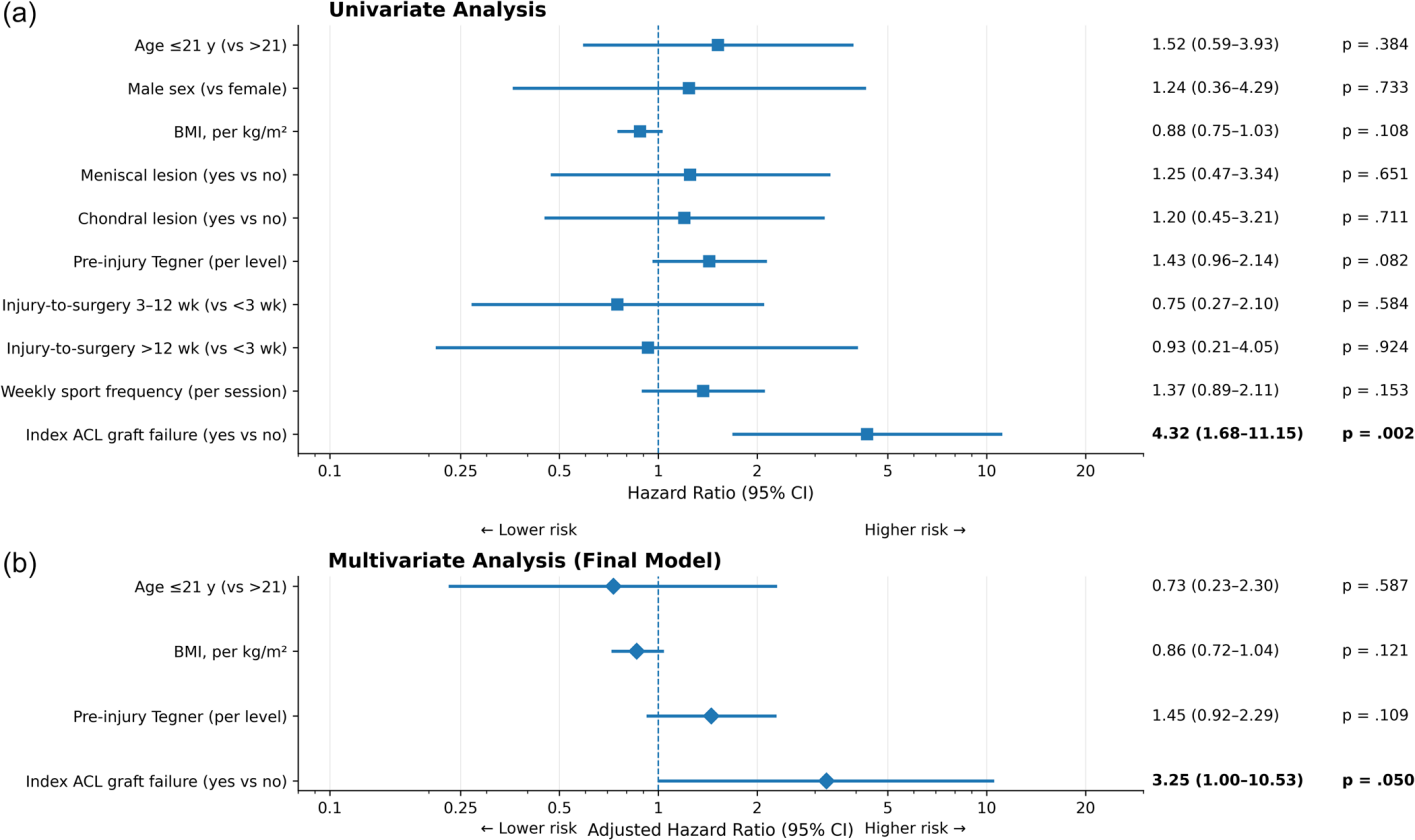
Forest plot of Cox regression analyses for risk factors associated with contralateral ACL injury after primary ACL reconstruction. (a) Univariate HRs and 95% CIs. (b) Exploratory multivariate model with aHRs and 95% CIs. The dashed vertical line indicates HR = 1.0. Bold text denotes statistically significant. Index ACL graft failure was modelled as a time‐varying covariate in the multivariate analysis. ACL, anterior cruciate ligament; aHRs, adjusted hazard ratios; BMI, body mass index; CI, confidence interval; HRs, hazard ratios.

When modelled as a time‐varying covariate, prior graft failure in the index knee was associated with contralateral ACL injury in univariable analysis (HR, 4.32; 95% CI, 1.68–11.15; *p* = 0.002) (Figure 5a). In the exploratory multivariable analysis adjusted for age, BMI and preinjury Tegner activity level, prior graft failure remained associated with contralateral ACL injury (adjusted HR, 3.25; 95% CI, 1.00–10.53; *p* = 0.050) (Figure 5b).

### Subjective evaluation and return to sport

Secondary functional outcomes at final follow‐up are summarized in Table 2. Overall, 381 patients (91.8%) returned to sport. Patients with intact grafts had higher SANE scores than patients with graft failure.

**Table 2:** Secondary functional outcomes at final follow‐up.

Domain	Outcome	Value
Return to sport	Returned to sport, *n*/*N* (%)	381/415 (91.8)
	Returned to same level among those who returned, *n*/*N* (%)	266/381 (69.8)
	Returned to same sport among those who returned, *n*/*N* (%)	247/381 (64.8)
Activity level	Postoperative Tegner activity level, median (IQR)	8 (6–8)
	Change from preinjury Tegner activity level, mean ± SD	−0.6 ± 1.1
Knee function	Single Assessment Numeric Evaluation score, mean ± SD	85.9 ± 11.9
	Single Assessment Numeric Evaluation score, median (IQR)	90.0 (80.0–90.0)
	Single Assessment Numeric Evaluation score, intact graft, mean ± SD	87.4 ± 9.6
	Single Assessment Numeric Evaluation score, graft failure, mean ± SD	76.5 ± 19.2
Self‐perceived sports performance	Worse/similar/better than preinjury, *n* (%)	311 (74.9)/99 (23.9)/5 (1.2)

Abbreviations: IQR, interquartile range; SD, standard deviation.

## DISCUSSION

In this retrospective cohort of 415 patients who underwent primary single‐bundle ACL reconstruction with hamstring tendon autograft, Kaplan–Meier graft survival was 93.3% at 2 years, 88.7% at 5 years and 86.6% at 10 years, whereas contralateral ACL survival was 98.8%, 97.1% and 95.5% at the same time points. In multivariable analysis, age 21 years or younger (adjusted hazard ratio [HR], 2.62) and concomitant chondral lesions (adjusted HR, 0.29) were associated with graft failure. In the exploratory multivariable analysis for contralateral ACL injury, prior graft failure in the index knee, modelled as a time‐varying covariate, remained associated with contralateral injury (adjusted HR, 3.25). These findings provide clinically relevant long‐term estimates for patient counselling after primary ACL reconstruction.

The 10‐year graft survival probability of 86.6% observed in the present cohort is consistent with the range reported in previous long‐term studies after primary ACL reconstruction, although direct comparisons should be interpreted considering differences in graft type, patient selection, activity profile and outcome definitions. In a population‐based study of 1019 patients with 10‐year follow‐up, Schilaty et al. [28] reported graft reinjury in 13.8% of patients. Bourke et al. [4], in a cohort of 755 patients followed for a minimum of 15 years, reported graft survival probabilities of 95%, 93% and 91% at 2, 5 and 10 years, respectively. The slightly lower 5‐year survival probability in the present cohort may partly reflect the prespecified worst‐case outcome definition, which classified patient‐reported characteristic ACL injuries without clinical or imaging confirmation as failures and may therefore have captured events not identified in registry‐based studies limited to documented revision surgery. In addition, the absence of extra‐articular stabilization techniques during the study period may have contributed to higher failure rates in the high‐risk subgroup [10]. A comparison of the present cohort with selected long‐term studies is provided in Table 3.

**Table 3:** Long‐term graft failure and contralateral ACL outcomes after primary ACL reconstruction—selected cohort and registry studies.

First author, year	Study design, *N* and population	Graft type and follow‐up	Definition of graft failure	Ipsilateral graft failure rate/survival	Contralateral ACL injury rate	Key factors: Graft failure and contralateral ACL injury
Sanders et al. 2017 [26]	Retrospective population cohort; *n* = 1355; general population (Olmsted County, MN); mixed age	Mixed (HT, BPTB, allograft) Mean FU 10 years; survival to 25 years	Ipsilateral graft failure after ACLR, chart‐confirmed	5.3% (72/1355); survival: 96% at 5 years, 94% at 10 years, 91% at 25 years	Not reported	Age ≤22 years (6.3% vs. 4.6%, *p* = 0.04); failure rate declined over 21‐year study period
Grassi et al. 2020 [12]	Prospective single‐centre cohort; *n* = 244; mean age 30.7 years; consecutive patients (Rizzoli Institute, Italy)	Hamstring autograft (four‐strand) + lateral plasty; min FU 10 years	Composite endpoint: ipsilateral ACL revision OR contralateral ACL reconstruction (both = second‐injury events); no imaging‐only definition	Ipsilateral revision: 3.4% (8/244); 10‐year ipsilateral survival 96.6%	7.8% (19/244) contralateral ACLR. From Year 6: HR 2.4–3.6 for contralateral versus ipsilateral revision. Young + active subgroup: 40% second ACL event at 10 years	Graft failure: no independent predictor identified. Contralateral: age <18 years; preoperative Tegner ≥7
Lindanger et al. 2019 [15]	Retrospective single‐centre cohort; *n* = 217 respondents (93%); pivoting‐sport athletes (handball, basketball, soccer); Norway	BPTB autograft; median FU 25 years (range 22–30)	Revision surgery to index knee (surgery‐confirmed); contralateral ACL injury by questionnaire	11% revision in returnees to preinjury sports level; 41% pooled reinjury (revision + contralateral) among returnees	30% contralateral ACL in returnees versus 4% in non‐returnees (*p* = 0.017); females 32%, males 23% (NS)	Graft failure + contralateral: return to preinjury pivoting sports (6.5× contralateral risk vs. non‐returnees). Late ACLR: higher knee replacement risk (9% vs. 3%)
Webster and Feller, 2016 [36]	Retrospective cohort; *n* = 316 analysed (89%); young athletes <20 years at ACLR; Melbourne, Australia	Hamstring autograft (4‐strand) Mean FU 5 years (range 3–10)	Graft rupture = rerupture of reconstructed ACL (contralateral native ACL documented separately)	18% overall (57/316); 28.3% in males <18 years; 12.9% in females <18 years; 74% of ruptures occurred within 2 years of surgery	17.7% (56/316) contralateral ACL injury. No significant age or sex difference for contralateral	Graft failure: younger age (<18 years); male sex (males <18 years at highest risk) Contralateral: no age or sex predictor identified
Rahardja et al. 2020 [23]	Prospective national registry (NZ ACL Registry); *n* = 7,155 (HT 77.7%, BPTB 22.3%); all‐comers primary ACLR 2014–2018	HT versus BPTB autograft Midterm FU (survival analysis; median FU not stated)	Revision ACLR and contralateral ACL reconstruction, both surgery‐confirmed via registry	HT 2.7% versus BPTB 1.3% revision aHR 2.51 for HT versus BPTB (95% CI 1.55–4.06, *p* < 0.001) NNT with BPTB to prevent 1 revision = 73.6	BPTB: higher contralateral risk aHR 1.91 versus HT (95% CI 1.15–3.16, *p* = 0.012); NNT with HT to prevent 1 contralateral = 116.3	Graft failure: HT graft (aHR 2.51 vs. BPTB) Contralateral: BPTB graft (aHR 1.91 vs. HT) Key message: BPTB reduces ipsilateral risk but shifts burden to contralateral knee
Randsborg et al. 2022 [24]	Institutional registry cohort (HSS, New York); *n* = 2042 enroled; 1045 respondents (51.2%); mixed graft/allograft practice	Mixed: BPTB 62%, HT 38% (autograft 76%); allograft 24%. Mean FU 7.2 years (5.0–9.8); revision analysis at 9 years	Revision ACLR (registry‐confirmed); contralateral ACLR recorded as separate outcome	7.2% revision at 9 years; 13% subsequent non‐ACL ipsilateral surgery. Graft choice did not predict revision or PROMs	6% contralateral ACLR; 26% chance of any additional knee surgery within 9 years	Graft failure: younger age; male sex; absence of meniscal tear at index surgery (independent in multivariate model); graft choice NS. Contralateral: not specifically analysed
Fältström et al. 2021 [8]	Prospective cohort; *n* = 222 respondents (70%); female soccer players; mean age 20.1 years; Sweden (multicenter)	Mixed/not specified (questionnaire FU; graft types not stratified); mean FU 6.5 years (range 5–10)	New ACL injury = ipsilateral rerupture OR contralateral ACL rupture; knee surgery recorded separately	Returnees: 44 reruptures (~27% of 163). Non‐returnees: 9 reruptures (~15% of 59)	Returnees: 29 contralateral ruptures (~18% of 163); 42% total new ACL in returnees; 68% had any new knee injury	Graft failure + contralateral: return to soccer (RR 2.24 vs. non‐return; RR 3.93 vs. knee‐healthy controls). Highest total new‐ACL burden in table; return‐to‐sport is dominant modulator

Abbreviations: ACL, anterior cruciate ligament; ACLR, anterior cruciate ligament reconstruction; aHR, adjusted hazard ratio; BPTB, bone–patellar tendon–bone; CI, confidence interval; FU, follow‐up; HSS, Hospital for Special Surgery; HT, hamstring tendon; NNT, number needed to treat; NZ, New Zealand; OR, odds ratio; PROMs, patient‐reported outcome measures.

The temporal pattern of graft failure observed in the present cohort is also consistent with prior reports showing that the first 2 postoperative years represent the period of greatest risk [1, 4, 7, 17, 19]. Fifty‐one percent of all graft failures occurred within the first 24 months after reconstruction. In contrast, contralateral ACL injury was observed later, with more than half of events occurring between 1 and 4 years after surgery. These findings suggest that the timing of ipsilateral graft failure and contralateral injury may differ and may have implications for the duration and focus of postoperative surveillance.

The association between younger age and graft failure is well established [1, 7, 22, 25, 34] and was confirmed in the present study. Patients aged 21 years or younger had a 2.62‐fold higher adjusted hazard of graft failure than older patients. This magnitude of association is consistent with registry‐based data and systematic reviews showing substantially higher revision rates in younger patients [22], and with cohort studies demonstrating an 8%–9% decrease in failure risk per additional year of age [13, 17]. Younger age likely reflects greater exposure to pivoting sports, higher activity demands and possibly earlier return to high‐risk activities. These findings support age‐specific counselling after ACL reconstruction and may be relevant when considering additional strategies to reduce failure risk in younger patients. Although more than half of the cohort underwent reconstruction within 3 weeks of injury, time from injury to reconstruction was not associated with graft failure in the present analysis.

Rather than implying a true protective biological effect, the present data suggest that chondral lesions may identify a subgroup with lower subsequent exposure to reinjury risk. Interpretation of other intra‐articular factors should be cautious, because standardized preoperative laxity data were not consistently available and meniscal pathology was captured only as a binary variable, without standardized documentation of tear type or treatment.

Contralateral ACL injury occurred in 4.3% of patients, with a 10‐year survival probability of 95.5%. In the exploratory multivariable model, prior graft failure in the index knee was associated with subsequent contralateral ACL injury. This association has been reported previously [5, 16, 30, 31, 35] and may reflect shared intrinsic or behavioural risk factors that predispose patients to bilateral ACL injury, including joint laxity, notch morphology, neuromuscular deficits or return to high‐risk sporting activities. However, this finding should be interpreted with caution because only 18 contralateral injuries occurred, resulting in limited precision and a wide CI.

Sex was not associated with graft failure or contralateral ACL injury in the present cohort, which is in line with the inconsistent findings reported in the literature [2, 3, 20, 21, 32]. The predominance of male patients in the present cohort may have limited the ability to detect sex‐based differences.

Functional outcomes at a median follow‐up of 10.6 years were generally favourable. Overall, 91.8% of patients returned to sport, 69.8% returned to the same level of activity and 64.8% returned to the same sport. The mean SANE score was 85.9 ± 11.9, and patients with intact grafts reported better knee function than those with graft failure. These results should be interpreted in the context of a predominantly recreationally active cohort and the use of patient‐reported follow‐up obtained at a single time point, which does not capture changes in sports participation over time [14, 18, 29, 37].

This study has several strengths. First, the cohort was sampled from a defined institutional database using simple random sampling, which may reduce selection bias compared with convenience sampling. Second, the analytical cohort was restricted to primary single‐bundle hamstring tendon autograft reconstruction, reducing heterogeneity related to graft type and surgical technique. Third, the follow‐up duration was long, with a median of 10.6 years, allowing assessment of both early and late events. Finally, the use of time‐to‐event methods allowed estimation of survival probabilities over time rather than relying only on crude proportions.

## LIMITATIONS

This study has several limitations. Graft failure and contralateral ACL injury were not assessed using systematic in‐person examination or imaging at final follow‐up. As a result, some subclinical laxity, partial graft failures or asymptomatic re‐ruptures may have been missed, whereas some patient‐reported events classified as failures under the prespecified worst‐case approach may not have represented true structural failure. Although this inclusive definition may have overestimated true structural failure in some cases, it was intended to reduce underascertainment of clinically meaningful late events in this long‐term retrospective cohort. Preinjury Tegner activity levels were assessed retrospectively at the time of telephone follow‐up, introducing the possibility of recall bias. In addition, 96 selected patients could not be reached or declined participation and were replaced using the randomized contact list; although this preserved the sampling strategy, the outcomes of nonresponders are unknown and may have differed from those of included participants. The single‐centre design and restriction to hamstring tendon autograft improve internal consistency but may limit generalizability to other settings, graft choices or surgical techniques. The contralateral ACL injury model should also be considered exploratory because only 18 events occurred. Furthermore, standardized preoperative laxity data were not consistently available across the historical cohort and therefore could not be analysed reliably. Meniscal pathology was available only as a binary variable for the full cohort; detailed meniscal treatment and tear‐pattern data were not collected in a standardized fashion, and meniscal lesions were heterogeneous in type and severity, which limited more granular analysis. Fixation methods were individualized rather than protocolized, precluding robust comparison according to fixation construct, and lower‐limb dominance was not documented consistently enough for reliable analysis. Finally, a small proportion of data were missing for some covariates, and complete‐case analysis was used for regression modelling.

## CONCLUSION

At a median follow‐up of 10.6 years after primary single‐bundle hamstring autograft ACL reconstruction, graft survival was 86.6% and contralateral ACL survival was 95.5%. Patients aged 21 years or younger had a substantially higher hazard of graft failure, and graft failure in the index knee was associated with subsequent contralateral ACL injury. For clinical practice, these findings indicate that younger patients should be counselled as a higher‐risk group not only for graft failure but also for future injury to the opposite knee.

## AUTHOR CONTRIBUTIONS

**Diego Alarcón Perico**: Conceptualization; study design; data collection and analysis; manuscript writing; and final approval of the paper. **Jorge Rojas Lievano**: Data analysis; statistical analysis; data interpretation; manuscript writing; critical revision of the manuscript and final approval of the paper. **Sergio González**: Literature review; patient recruitment and data collection; manuscript preparation; and final approval of the paper. **Gamal Zayed**: Conceptualization; study design; data interpretation; critical revision of the manuscript; and final approval of the paper. **German Carrillo Arango**: Conceptualization; study design; data interpretation; critical revision of the manuscript; and final approval of the paper. **Klaus Mieth Alviar**: Study supervision; project administration; study design; data interpretation; critical revision of the manuscript; final approval of the paper.

## FUNDING INFORMATION

The authors have no funding to report.

## CONFLICT OF INTEREST STATEMENT

Each author certifies that he has no commercial associations (e.g., consultancies, stock ownership, equity interest, patent/licensing arrangements) that might pose a conflict of interest in connection with the submitted article.

## ETHICS STATEMENT

This study was approved by Hospital Universitario Fundación Santa Fe de Bogotá (approval number: CCEI‐13122‐2021). This study was approved by the Institutional Review Board of [IRB CCEI‐13122‐2021]. Informed consent was obtained from all individual participants at the time of the follow‐up telephone assessment. For the retrospective review of institutional medical records, a waiver of informed consent was granted by the ethics committee, as the data were de‐identified and analysed anonymously.

## Data Availability

The data that support the findings of this study are available on request from the corresponding author. The data are not publicly available due to privacy or ethical restrictions.
